# Human V6 Integrates Visual and Extra-Retinal Cues during Head-Induced Gaze Shifts

**DOI:** 10.1016/j.isci.2018.09.004

**Published:** 2018-09-08

**Authors:** Andreas Schindler, Andreas Bartels

**Affiliations:** 1 Vision and Cognition Lab, Centre for Integrative Neuroscience, University of Tübingen, Otfried-Müller-Str. 25, Tübingen 72076, Germany; 2 Department of Psychology, University of Tübingen, Tübingen 72076, Germany; 3 Max Planck Institute for Biological Cybernetics, Tübingen 72076, Germany; 4 Centre for Integrative Neuroscience & MEG Center, University of Tübingen, Tübingen 72076, Germany

**Keywords:** Neuroscience, Sensory Neuroscience, Techniques in Neuroscience

## Abstract

A key question in vision research concerns how the brain compensates for self-induced eye and head movements to form the world-centered, spatiotopic representations we perceive. Although human V3A and V6 integrate eye movements with vision, it is unclear which areas integrate head motion signals with visual retinotopic representations, as fMRI typically prevents head movement executions. Here we examined whether human early visual cortex V3A and V6 integrate these signals. A previously introduced paradigm allowed participant head movement during trials, but stabilized the head during data acquisition utilizing the delay between blood-oxygen-level-dependent (BOLD) and neural signals. Visual stimuli simulated either a stable environment or one with arbitrary head-coupled visual motion. Importantly, both conditions were matched in retinal and head motion. Contrasts revealed differential responses in human V6. Given the lack of vestibular responses in primate V6, these results suggest multi-modal integration of visual with neck efference copy signals or proprioception in V6.

## Highlights

• Setup with head-mounted goggles and head movement during fMRI
• Simulation of forward flow in stable or unstable world during head rotation
• Human V6 integrates visual self-motion with head motion signals
• Likely mediated by efference copy or proprioception as V6 lacks vestibular input

## Graphical Abstract

**Figure:**
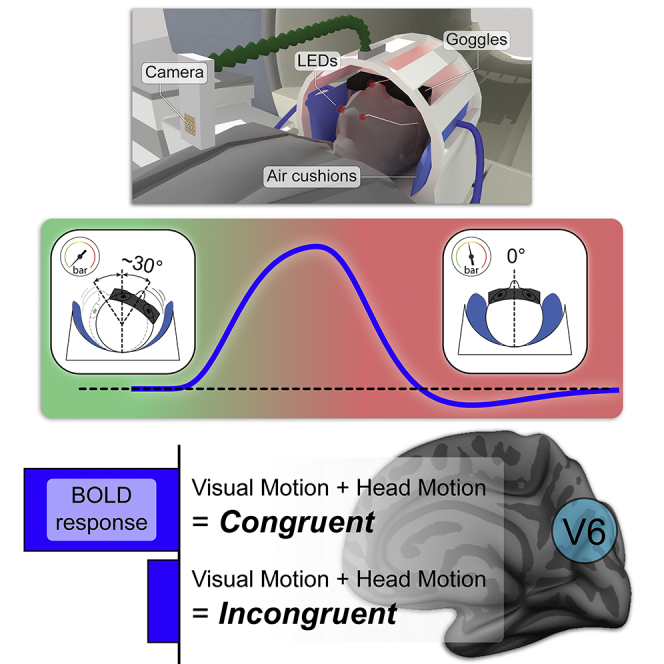

## Introduction

A remarkable property of the visual system is its feat to provide us with stable vision despite continuously changing retinal input induced by our movements of eyes, head, and body. This feat appears especially intriguing as the majority of visual areas are organized retinotopically, yet stability requires integration of visual input with cues from other modalities. Although the integration of eye movements with retinal signal has been studied extensively in both monkeys (Galletti et al., 1984, Galletti et al., 1988, Galletti et al., 1990, Erickson and Thier, 1991, Ilg et al., 2004, Dicke et al., 2008) and humans (Goossens et al., 2006, Arnoldussen et al., 2011, Fischer et al., 2012, Nau et al., 2018), the integration of visual signal with voluntary head movements remains barely studied at the level of neocortex (see Carriot et al., 2013, Cullen and Taube, 2017 for subcortical function).

In macaques and humans, previous studies examining cortical function focused on passive head motion or artificial vestibular stimulation to examine visual-vestibular integration (Gu et al., 2008, Chen et al., 2011, Smith et al., 2012, Frank et al., 2014, Frank et al., 2016, Billington and Smith, 2015). However, active gaze shifts beyond eye movements also involve head rotation (Land, 1992). In fact, gaze change commands reach eye and head effector muscles at the same time (Bizzi et al., 1971), and human observers compensate for eye- and head-induced self-motion with equal precision (Crowell et al., 1998). Notably, however, despite this prominent role for head motion in visual stability almost nothing is known about which visual processing stages integrate head motion signals with retinotopic representations as technical limitations have hindered human neuroimaging to study the neural underpinnings of voluntary head movements.

**Figure 1:**
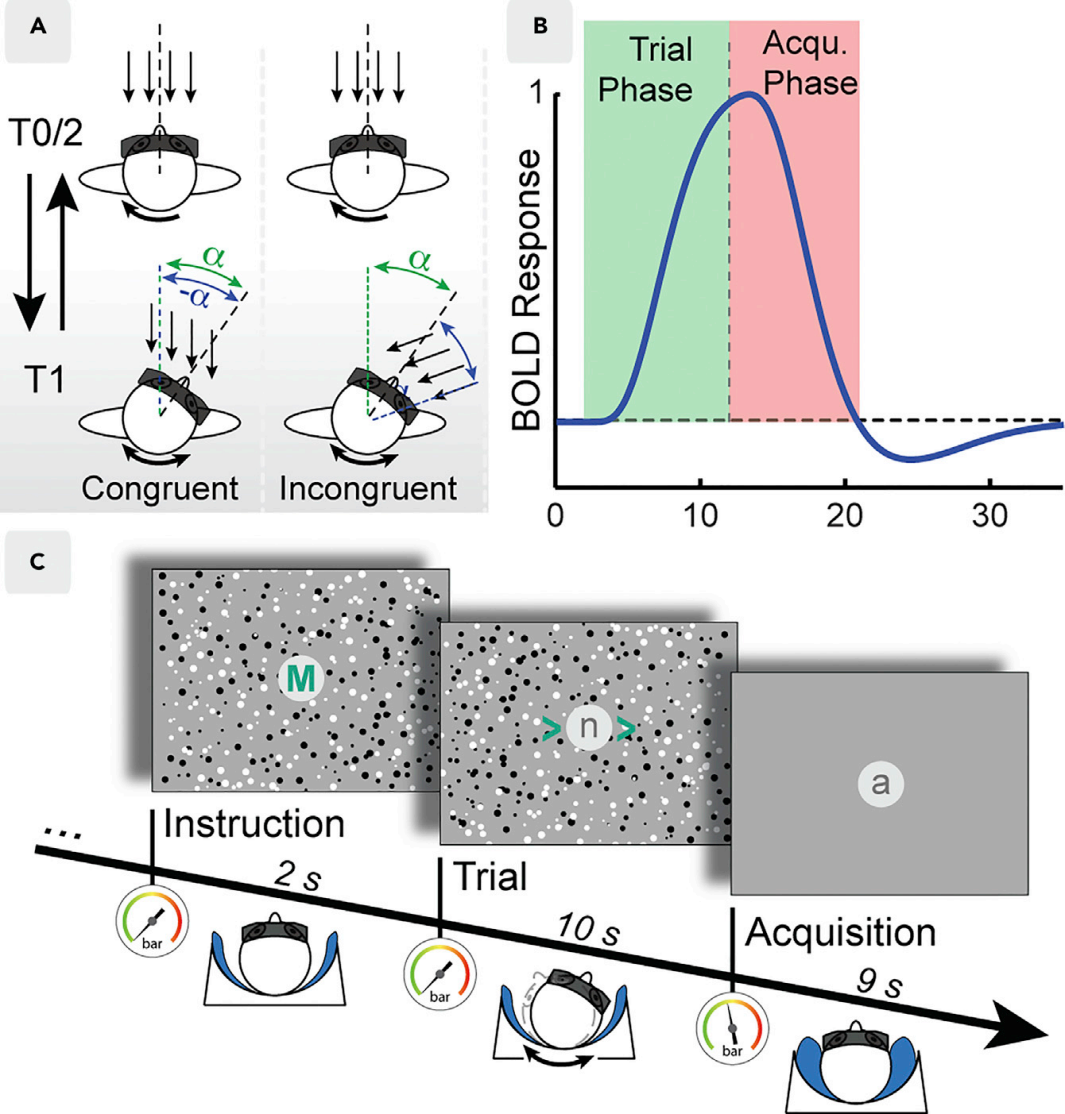
Illustration of Visual Stimuli and Head-Rotation Task, BOLD Signal Acquisition during a Trial, and Experimental Paradigm (A) Observers performed voluntary head rotations while being approached by a simulated 3D dot cloud in both congruent and incongruent conditions. Head rotations in the congruent condition lead to cloud rotation in opposite direction (-α) to the observer's head (α), as would be experienced when moving forward in a stable environment and looking around. In the incongruent condition, the cloud and head rotated in the same direction (α), resulting in perceptually arbitrary motion of the environment. Note that retinal flow as well as head motion were matched in both conditions. (B) Model of the evoked BOLD time course as predicted by the paradigm in (C). Stimulus presentation and active head movements induced BOLD signals during the trial phase (green shade) while the slow dynamics of the BOLD signal allowed acquisition of these responses even after stimulus offset, at a time when the observer's head was stabilized (acquisition phase, red shade). (C) Each trial started with an instruction phase when air cushions were emptied. In the trial phase, green arrowheads guided the observer's head rotation. In the acquisition phase, air cushions were inflated again to record BOLD responses. Observers performed a demanding fixation task across all phases and conditions, except the instruction phase (see Methods).

We recently circumvented these limitations and introduced an approach that allows participants to move their heads during fMRI scanning by exploiting the delay of several seconds between neural processing and blood-oxygen-level-dependent (BOLD) signal (see Figures 1A–1C) (Schindler and Bartels, 2018). We constructed a custom-built air pressure-based head stabilization system that permitted head rotation during trials, but stabilized head position during data acquisition. Observers wore head-mounted magnetic resonance-compatible goggles while head movement was tracked online. This allowed generation of visual stimuli that could be modulated by head motion in real time (Schindler and Bartels, 2018). In two conditions, observers viewed approaching visual flow that was modulated by head motion. A congruent condition simulated a scenario of constant forward motion where head rotation resulted in looking around while being driven along a straight road. In the incongruent condition, observers performed identical head rotations, but visual consequences of head rotation were inversed such that visual and extra-retinal cues did not combine in any meaningful way but retinal motion was matched to the congruent condition. In both conditions, a demanding letter detection task assured fixation. Based on this paradigm we previously examined the integration of head movements and visual signals in a network of areas with established vestibular input. Particularly, a contrast between congruent and incongruent conditions revealed evidence consistent with the multi-modal integration of visual cues with head motion into a coherent “stable world” percept in the parietal operculum and in the anterior part of the parieto-insular cortex. This also applied for a subset of visual motion-responsive areas such as human medial superior temporal area (MST) (at uncorrected level), the dorsal part of the ventral intraparietal area (VIP), the cingulate sulcus visual area (CSv), and a region in the precuneus (Pc) (Schindler and Bartels, 2018). However, the important question whether retinotopic cortex and especially areas V3A and V6 play a role in visual stability during voluntary head movement remained open.

## Results

We here fill this gap by combining the previously collected dataset with newly acquired retinotopic localizer data in the same participants for early visual areas V1, V2, V3, and V4 as well as higher visual areas V3A and V6 (see Methods). We probed each area using the BOLD contrast between congruent and incongruent conditions. Notably, these conditions were matched in both, head motion and retinal visual flow. However, only the congruent condition reflected a stable world and hence a match between visual and extra-retinal signals, whereas the incongruent condition constituted an arbitrary combination of both features. We thus operationalized “visual stability”—i.e., to what extent a given visual area might integrate its retinotopic input with extra-retinal signals—as differential BOLD activity to this contrast. Although we used this measure to probe a given area for integration, we had no a priori hypothesis regarding the sign of this difference. In fact, given the lack of the underlying integration mechanism as well as the complex relation between neural activity and BOLD signals, many scenarios are conceivable (Logothetis et al., 2001, Logothetis and Wandell, 2004, Goense and Logothetis, 2008, Logothetis, 2008).

**Figure 2:**
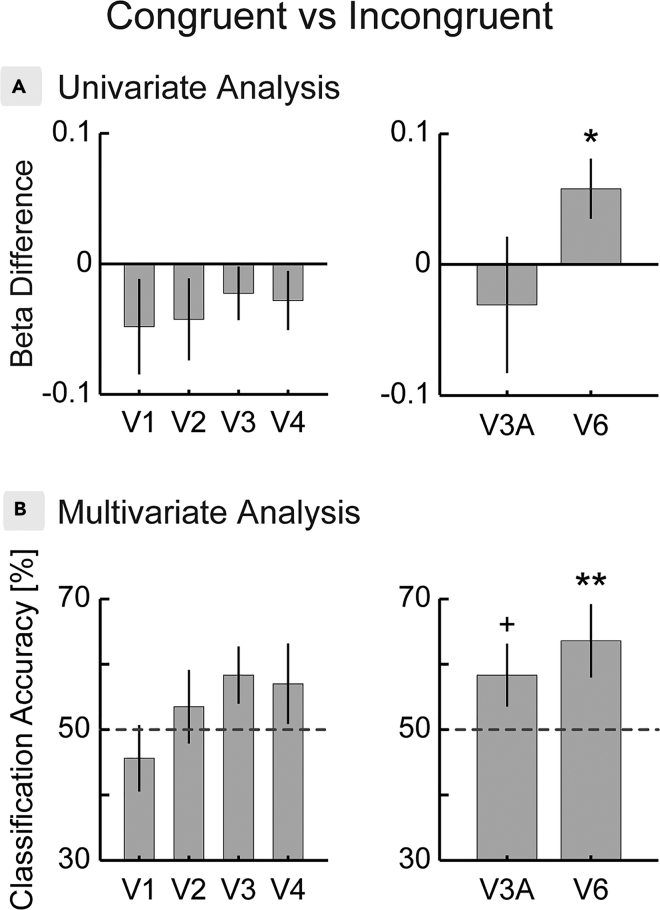
Univariate and Multivariate Differences between Congruent and Incongruent Combinations of Visual and Extra-Retinal Signals in Visual Cortex (A) A contrast between both conditions revealed significant responses to the congruent combination of both cues in area V6, whereas V3A and early visual areas showed no differential activation. (B) A subsequent multivariate pattern analysis found robust classification of both conditions in area V6, whereas effects in V3A did not survive multiple comparison correction. Patterns in early visual cortex did not distinguish between congruent and incongruent conditions. Dashed lines indicate chance level. ^+^p < 0.05, uncorrected; *p < 0.05, FWE-corrected; **p < 0.005; FWE-corrected. Error bars indicate SEM. See Figure S1 for responses to an additional “head only” condition and Figure S2 for retinotopic area definitions of example observers.

Neither BOLD responses in early visual cortex (V1: T(18) = −1.32; p = 0.771; V2: T(18) = −1.35; p = 0.771; V3: T(18) = −1.10; p = 0.771; V4: T(18) = −1.24; p = 0.771; corrected for family-wise error (FWE) nor activation in V3A (T(18) = −0.60; p = 0.559; FWE-corrected) distinguished between congruent and incongruent conditions (Figure 2A). We found, however, significantly increased responses to the congruent combination of visual and extra-retinal signals in area V6 (T(18) = 2.52; p = 0.043; FWE-corrected) (Figure 2A). To reexamine these results, we performed a multivariate pattern analysis training classifiers to distinguish congruent and incongruent conditions based on multi-voxel region of interest (ROI) patterns. Using an odd/even cross-validation approach we trained support vector machines to distinguish both conditions on one-half of the data and tested them on the other half. Notably, this analysis confirmed our positive result in V6 (mean accuracy = 0.64 ± 0.06; p = 0.004; FWE-corrected) and the lack of differential activity in the early visual cortex (V1: mean accuracy = 0.46 ± 0.05, p = 0.987; V2: mean accuracy = 0.54 ± 0.06, p = 0.548; V3: mean accuracy = 0.58+-0.04, p = 0.126; V4: mean accuracy = 0.57 ± 0.06, p = 0.217; FWE-corrected). In addition, it revealed a small effect in V3A (mean accuracy = 0.58 ± 0.05, p = 0.024, uncorrected; p = 0.101, FWE-corrected) that did not survive correction for multiple comparisons.

We took great care to rule out alternative accounts of our results. There were no differences in fixation task performance between congruent and incongruent conditions, and quantitative analysis of head movements as well as visual motion energy showed that both were matched between conditions (Schindler and Bartels, 2018).

## Discussion

Despite substantial progress in vision research, the mechanisms of how transient retinotopic representations may give rise to stable vision are far from being understood (Burr and Morrone, 2011). In particular, evidence for the compensation of visual self-motion induced by voluntary head motion is scarce. Based on a recently established paradigm (Schindler and Bartels, 2018) we here provide first evidence for the integration of retinotopic representations and voluntary head movement signals in the human homolog of area V6.

Already early studies in macaques found strong visual motion selectivity in V6 (Galletti et al., 1996, Galletti et al., 1999). These findings are paralleled by a growing body of human neuroimaging studies that point to human V6 as a prominent visual motion area specialized in the processing of self-motion flow fields (Cardin and Smith, 2010, Pitzalis et al., 2010, Arnoldussen et al., 2011, Cardin et al., 2012b, Fischer et al., 2012, Schindler and Bartels, 2017). However, the role that self-motion signals in V6 may play has been an open question. Monkey electrophysiology found visual heading selectivity in V6 neurons (Fan et al., 2015), whereas human V6 shows no adaptation effects when presented with successive optic flow patterns (Cardin et al., 2012a). In fact, the lack of BOLD and single cell responses to vestibular signals in V6 of man (Smith et al., 2012) and monkey (Fan et al., 2015) even further deemphasize a role for V6 in heading perception. Rather, V6 has been suggested to play a major role in the compensation of self-induced visual motion to compute object motion. In line with this, “real motion” neurons in V6 receive efference copies of eye movements and attenuate or stop firing when retinal motion is induced by active pursuit over a static target (Galletti and Fattori, 2003). In fact, human V6 even shows near-complete multi-modal integration of planar visual and non-visual motion signals during pursuit (Fischer et al., 2012). Corroborating the self-motion compensation view, our results provide evidence for the integration of self-motion-compatible visual flow signals with non-retinal voluntary head motion signals in human V6. Although the integration of retinotopic information and eye movements is predominantly mediated by corollary discharge (Sommer and Wurtz, 2002, Cavanaugh et al., 2016), less is known about integration signals of voluntary head movements. The lack of passive inertial head motion signals in monkey V6 (Fan et al., 2015), together with the absence of responses to galvanic vestibular stimuli in human V6, render it likely that the integration of visual and extra-retinal head motion signals observed here may have been driven by efference copy signals or proprioception. Given the self-motion hypothesis of V6, this idea is further corroborated by the fact that proprioceptive information in combination with neck efference copy signals are sufficient to allow human observers accurate compensation for head-induced visual self-motion, whereas vestibular cues presented in isolation are not (Crowell et al., 1998). Following these arguments, we appreciate the sources of extra-retinal signals as an important difference between the present results in V6 and our previous findings concerning the integration of visual and head motion signals in areas with prominent vestibular responses. Although these included the posterior insula and human VIP, integration responses in human MST reached significance only at the uncorrected level, whereas area MT/V5 showed no differential responses to either condition (Schindler and Bartels, 2018). Nevertheless, monkey V6 as well as its human homolog form major connections to both (h)MST and (h)VIP (Galletti et al., 2001, Tosoni et al., 2015). In fact, all these areas have been found to contain neurons that code visual space in craniotopic coordinates (Galletti et al., 1993, Duhamel et al., 1997, Colby and Goldberg, 1999, Ilg et al., 2004). Notably, however, in these experiments the monkey's head was typically restrained such that head-centered and body-centered coordinates coincided. Against this background, the present results render it likely that, at least to some extent, craniotopic responses in human V6 (in addition to hMST and hVIP [Schindler and Bartels, 2018]) may actually generalize to a body-centered reference system, as it has been proposed for other parietal regions (Brotchie et al., 1995). A body-centered reference framework would also well be in line with the notion that V6 plays a major role in mapping vision onto action (Galletti et al., 1999, Galletti et al., 2001, Pitzalis et al., 2012, Tosoni et al., 2015). This seems particularly evident for grasping as, directly adjacent to V6, cells in V6A are tuned to arm movements (Fattori et al., 2001) as well as to hand orientation (Fattori et al., 2009). In light of the present results, interactions of V6 and V6A could thus mediate visually guided arm movements during gaze shifts regardless of whether they are induced by eye or head movements. In addition, a body-centered reference frame is also consistent with a possible role for V6 in flow parsing for navigation as it has been suggested based on functional properties and prominent connections between V6 and para-hippocampal cortex in humans and monkeys (Galletti and Fattori, 2003, Galletti and Fattori, 2018, Pitzalis et al., 2010, Tosoni et al., 2015).

In contrast to V6, evidence for the integration of head movement and retinotopic signals in human V3A was only evident in a more sensitive multivariate pattern analysis (MVPA) analysis and did not survive corrections for multiple comparisons (see Figure 2B). Given the prominent role of V3A in the integration of eye movements with visual signals, this finding seems counter-intuitive at first sight. However, the additive forward flow used in the present study had abolished the ability of V3A to compensate planar motion for eye movements previously, whereas V6 still compensated (Fischer et al., 2012). This suggests a more advanced flow parsing mechanism in V6 able to isolate planar motion from expansion flow. Additional studies are hence needed to carefully examine the possible role of V3A in the compensation for head-induced self-motion during planar flow alone. We also found no evidence for the integration of retinotopic signals and head movements in early visual cortex. As for V3A this does not mean that early visual areas might not integrate these signals in a different setting. However, the fact that responses there did not differentiate between congruent and incongruent conditions corroborates that both conditions were well matched in low-level features.

Considering the substantial body of literature on visuo-vestibular integration in monkeys (Gu et al., 2008, Chen et al., 2011), caloric and galvanic stimulation in human neuroimaging (Brandt et al., 1998, Smith et al., 2012, Frank et al., 2014, Frank et al., 2016, Billington and Smith, 2015), as well as the results of our previous study (Schindler and Bartels, 2018), evidence converges toward a network of cortical brain areas that may directly or indirectly be involved in maintaining our stable experience of the visual world while we navigate in it. This network includes not only regions of the posterior insula as central hubs of vestibular processing but also areas with vestibular input that were initially associated with visual (self-)motion processing such as MST, VIP, and CSv. Although this list is far from being complete, the present results point to V6 as an important node in this network concerned with the integration of visual and extra-retinal signals during head-induced gaze shifts. Given the apparent lack of vestibular input to this area, future studies are required to examine possible sources of head-driven signals that may enable such integration in V6.

### Limitations of the Study

As shown in our previous study, voluntary head motion and artificial vestibular stimulation both led to similar BOLD responses in insular cortex and areas of the self-motion network (Schindler and Bartels, 2018). However, a major advantage of voluntary head movements over artificial vestibular stimulation is the ecological validity, and foremost, the possibility to precisely match high-frequency temporal aspects and intensity between visual and non-visual signals and therefore provide an immersive, realistic experience. A disadvantage of head movements is that contributions of non-retinal signals, i.e., efference copy, and proprioceptive and vestibular signals coincide and thus cannot be disentangled. Although no previous evidence for vestibular signals in primate V6 exists (Smith et al., 2012, Fan et al., 2015), the present study thus cannot conclusively answer the question what source head signals for multi-modal integration in V6 may have.

## Methods

All methods can be found in the accompanying Transparent Methods supplemental file.

## Supplemental Information

Document S1. Transparent Methods and Figures S1 and S2
