# Supplementary material for: Human V6 Integrates Visual and Extra-Retinal Cues during Head-Induced Gaze Shifts
Source: iScience. 2018 Sep 8;7:191–7. doi: 10.1016/j.isci.2018.09.004 (PMC6153141; doi:10.1016/j.isci.2018.09.004)
Supplement: Document S1. Transparent Methods and Figures S1 and S2 [file mmc1.pdf]

ISCI, Volume 7

Supplemental Information

Human V6 Integrates Visual and Extra-Retinal Cues during Head-Induced Gaze Shifts

Andreas Schindler and Andreas Bartels

Supplemental Figures

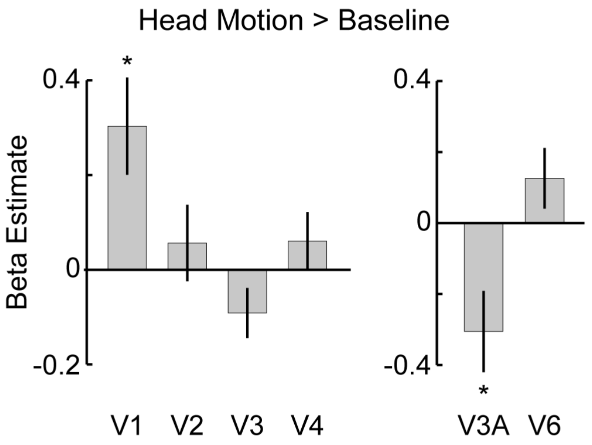

Figure S1 (relates to main figure 2): Head motion responses in visual areas. A contrast between the "head only" condition and a baseline condition involving static dots found differential BOLD responses in V1 and V3A while the others areas showed no differential activation. *= $p < 0.05$. FWE corrected. In an additional explorative analysis we compared responses to a "head only" condition that involved observer head motion while fixating a static dot display to a baseline condition involving "static" dots in the absence of head motion. Based on this analysis, we found differential responses in V1 while the remaining areas of early visual cortex showed no effects (V1: $T(18) = 2.95$; $p = 0.034$; V2: $T(18) = 0.70$; $p = 0.679$; V3: $T(18) = -1.73$; $p = 0.301$; V4: $T(18) = 0.98$; $p = 0.679$; FWE corrected). We additionally found BOLD modulation to "head only" in V3A, while V6 did not respond to head motion in the absence of additional visual motion (V3A: $T(18) = -2.67$; $p = 0.031$; V6: $T(18) = 1.47$; $p = 0.158$; FWE corrected). As described in the previous study (Schindler and Bartels, 2018), the "head only" condition was consistent with an additional interpretation. As the dot field was static with respect to the head (no dot motion in the head-mounted goggles) this condition was also consistent with world-centered object motion. Responses to this condition could thus have reflected object motion selectivity or rather head motion driven efference copy, proprioceptive or vestibular (even though less likely cf. (Smith et al., 2012)) signals in these areas. Scenarios for both, or even interactions between both stimulus interpretations are conceivable. While head motion was perfectly matched between congruent and incongruent conditions, there was a small (mean differences in translation: 0.01 mm ± 0.01 mm and rotation: 0.02° ± 0.00°) but significant ($p < 0.05$, FWE-corrected) difference in head motion between the "head only" and baseline conditions (see figure 6 in (Schindler and Bartels, 2018)). While we deem this influence as marginal given the fact that the affected contrast "head only" vs. "static" evoked opposing responses even in closely adjacent areas (i.e. V5/MT and MST; PIVC and pPIC, aPIC) previously (Schindler and Bartels, 2018), we cannot entirely exclude that this effect might have influenced results of this particular contrast in one of the areas examined here.

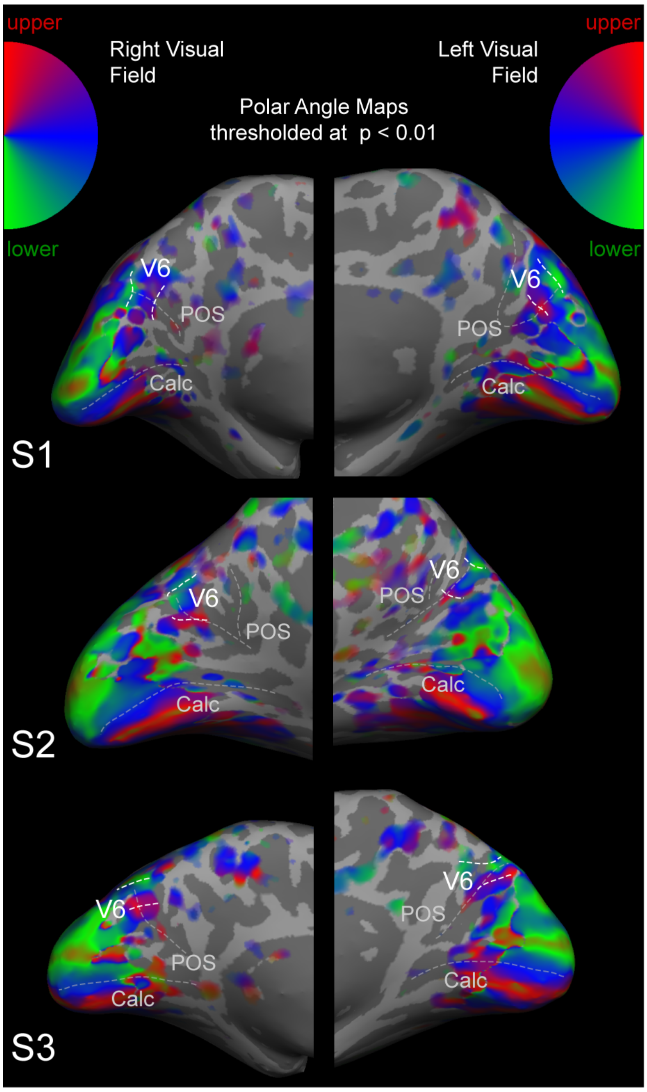

Figure S2 (relates to main figure 2): Retinotopic data of three example observers. Polar angle maps are thresholded at $p < 0.01$. Based on previous evidence (Pitzalis et al., 2006; Pitzalis et al., 2010; Pitzalis et al., 2012, 2015), V6 was defined as the contralateral hemi-field representation in the dorsal-most part of the parieto-occipital sulcus (POS).

Transparent Methods

Stimulus paradigm

The data used in the present study were acquired within a counterbalanced design involving a total of five conditions (Schindler and Bartels, 2018). For the purpose of the present study, two conditions were used for analysis. While a summary is given below, a full and comprehensive description of the paradigm, the customized hardware as well as all details of head motion and data analyses have been published previously (Schindler and Bartels, 2018).

In each condition, a trial comprised three phases. In the instruction phase (2 s), air cushions were emptied and a green "M" indicated that the next phase involved head rotation. The onset of the trial phase was indicated by the appearance of two green arrows to the left and right of the fixation dot. During the trial phase (10 s) the observer then moved the head as directed by the arrows, while the condition specific stimulus was presented. In the acquisition phase (9 s), air cushions were filled to record stimulus induced BOLD signals. Observers were instructed to continuously and slowly rotate their heads two times from center position (0°) to either ~+30° or ~ -30° and back. A given trial either involved rotations between 0° and ~-30° or between 0° and ~+30°, and trials of both directions were counterbalanced. Two green arrows guided observers' head rotations and switched orientation direction when the head reached a given angle threshold (i.e. 0°, 30°, −30°) based on online head tracking data. At the end of each head rotation cycle, both arrows disappeared and the fixation disc turned red until the observer's head reached center position again. In case the observer failed to complete the current rotation cycle, the fixation disc turned red after 10 s (i.e. after the entire trial phase) elapsed. Such trials were removed from further analysis. Subsequently, the acquisition phase started with filling of the air cushions.

Observers viewed stimuli via MR proof video goggles (resolution: 800 × 600; refresh rate: 60 Hz; VisuaStim, Resonance Technology Inc.) providing a visual field of 30 × 23 visual degrees. The paradigm was programmed using Psychtoolbox 3 (http://psychtoolbox.org/) and ran on MATLAB 2010 (Mathworks) on a Windows PC.

Trial phases of both conditions were based on random dot patterns that contained on average 400 black and white dots, presented on a gray (90 cd/m2) background at 100% contrast. Both conditions simulated a 3D dot cloud in front of the observer. The visibility of the cloud began at 360 cm and ended at 120 cm. Dots changed size with distance, subtending a maximal diameter of 2.2°. No disparity cues were provided. To study self-motion integration during simulated forward motion we added a translational component to dot motion such that dots approached the observer at a constant speed of 120 cm/s. Dot motion was modulated dependent on observers' head rotation using real-time feedback from the head-tracking camera. In the "congruent" condition the 3D dot cloud rotated in opposite direction relative to the observer's head. Notably, this condition allowed the observer to integrate visual self-motion motion cues and head movement into a coherent stable world percept, i.e. as experienced in the real world when a navigator rotates his/her head while traveling along a straight linear path. It hence allowed for integration of multimodal cues just as experienced in real-life scenarios. In contrast, head rotation in the "incongruent" condition involved rotation of the 3D dot cloud in the same direction as the observer's head. Thus, this condition provided identical visual motion energy (in retinal coordinates) and head motion as the "congruent" condition, but only provided arbitrary coupling of both cues in ways that cannot be encountered in real-world scenarios. In the "static" and "head motion" conditions each trial involved a single static projection of the 3D dot cloud, i.e. devoid of any dot motion. Note that since in the "head motion" condition the

dot field was static with respect to the head (no dot motion in the head-mounted goggles), this condition also involved world-centered object motion.
To ensure fixation, vigilance and balanced attention across all conditions, observers performed a demanding letter detection task during all conditions in trial and acquisition phases (but not during the instruction phase). The task was presented within the fixation disc (1.3° diameter) that was presented at the center of the head mounted display. Observers were asked to press a button whenever an alphabetical letter (a–z, size: 0.9°) with same identity appeared twice in a row. Letters appeared every 830 ms with repetitions occurring on average every 6 s.

Observers

A sub sample of 19 of the originally acquired 21 observers (Schindler and Bartels, 2018) took part in this study (8 males, 11 females, age 20–36 years; mean 27 years). The remaining two observers were no longer available at the time we acquired retinotopic data. All participating observers gave written informed consent prior to participation. The study was approved by the ethics committee of the University Clinic Tübingen.

Region of interest definition

Retinotopic maps of areas V1, V2, V3 and V4 as well as areas V3A and V6 were delineated in 19 observers using standard retinotopic mapping techniques (Sereno et al., 1995; Swisher et al., 2007). In contrast to the main experiment that used MR-compatible head-mounted goggles, retinotopic stimuli were back-projected using a projector onto a screen positioned behind the observers' head and viewed via a front-surfaced mirror mounted on the head coil, with 1280 × 1024 pixels resolution at 60 Hz. The visual display subtended 24 × 18 visual degree and was viewed at 80 cm distance. The stimulus consisted of a wedge shaped checkerboard with an arc angle of 90 degrees and a radius of 12 visual degrees (100% contrast, 6 Hz contrast inversion flicker, check sizes increased logarithmically with eccentricity) rotating around the central fixation dot (55.7 s period, matched clockwise and counterclockwise rotation) in front of a gray background. An attention task was superimposed onto the checkerboard to facilitate parietal responses (Swisher et al., 2007). For preprocessing, images were slice time corrected and smoothed by a Gaussian kernel of 4 mm full width half maximum. Polar angle maps were calculated as described in previous studies (Sereno et al., 1995), and projected on inflated brain surfaces of individual participants. This way, polar maps of occipital cortex were used to define areas V1, V2, V3 and V4. V3A was delineated as the intersection of the retinotopically defined V3AB-complex and a dedicated functional motion localizer. This localizer defined V3A as the strong response on the superior occipital lobe to pursuit with co-moving objective motion versus pursuit on a static background, corresponding to contrasting objective with retinal motion, which replicably leads to selective activation of voxels overlapping with retinotopically defined V3A (Fischer et al., 2012).
Based on previous evidence, we defined human area V6 as the contralateral hemi-field representation in the dorsal-most part of the parieto-occipital sulcus (POS) (Pitzalis et al., 2006; Pitzalis et al., 2010; Pitzalis et al., 2012, 2015) (see also example data in supplemental figure 2). We used this approach instead of a functional motion localizer for the delineation of V6 as the latter may have increased the risk to

incorrectly label voxels of the adjacent V6A as V6 (Pitzalis et al., 2015). In fact, V6 as well as V6A have both shown to respond to visual motion while only V6 has a prominent retinotopic representation including the center of gaze (Pitzalis et al., 2015). While definition of area V6 is facilitated using wide-field retinotopic stimuli (Pitzalis et al., 2006; Pitzalis et al., 2010), V6 has previously also been delineated using standard retinotopic procedures (Pitzalis et al., 2010; Cardin et al., 2012b; Fischer et al., 2012). We here used retinotopic stimuli comparable in size with the main flow stimuli to assure that the selected V6 voxels were also stimulated by the main optic flow stimuli. Surface reconstruction (Dale et al., 1999), analysis of polar angle maps as well as retinotopic ROI delineation was done in FreeSurfer (http://surfer.nmr.mgh.harvard.edu/).

MRI acquisition

Functional gradient-echo echoplanar T2-weighted images (EPI) were acquired on a Siemens 3T Prisma scanner with a 20-channel phased-array head coil (Siemens, Erlangen, Germany), with the following parameters: TR 754 ms, TE 30 ms, flip angle 55°, field of view 192 × 192 mm, multiband factor 3. Images consisted of 36 slices with 64 × 64 pixels (3 mm thick, no gap), resulting in 3 × 3 × 3 mm voxels. Slices were acquired in an interleaved fashion. We recorded six runs of the main experiment each consisting of 1415 images (including all 5 conditions initially acquired). This involved images for all three experimental phases of all trials. Motion localizer and retinotopy data were acquired with a higher resolution of 2 × 2 × 2 mm in 56 slices (TR 870 ms, TE 30 ms, flip angle 56°, GRAPPA factor 2 and multiband factor 4) using a 64 channel head coil. One run of the motion localizer consisted of 710 images. One run of polar wedge retinotopy involved 660 images. We acquired one or two runs motion localizer and four runs of retinotopy per observer, respectively. For each run, the initial eight images were discarded to allow for equilibration of T1 signal. A high-resolution anatomical scan was also obtained for each observer with a T1-weighted MP-RAGE sequence of 1 × 1 × 1 mm resolution.

Data Analysis

The central steps of the fMRI analysis are provided in the following, as the full description has been provided elsewhere (Schindler and Bartels, 2018). Only images that were recorded during the acquisition phase (see figure 1B) were considered for further analysis. Preprocessing involved rejection of head-motion artifacts, followed by preprocessing using SPM12 (http://www.fil.ion.ucl.ac.uk/spm/) including head-motion correction and spatial normalization to MNI space. Images were spatially smoothed using a Gaussian Kernel of 6 mm full-width at half maximum.

Each observer was analyzed separately using a GLM. We initially built the full design matrix based on the entire time series including time points that were affected by motion artifacts. Thus, each of the five initially acquired conditions was modeled separately and was convolved with the hemodynamic response function as implemented by SPM12. To account for potentially remaining head movement related variance and for drifts, a set of nuisance regressors was included. For model estimation we removed all time points affected by motion artifacts from design matrix and time series. This procedure maintained the temporal structure of data and model despite the removal of artifact-polluted images.

For ROI analyses, condition betas for congruent and incongruent conditions were extracted in each observer individually after ROIs had been projected to standard space. ROIs of both hemispheres in a given observer were averaged for each visual area. Significant differences between conditions were assessed using one-sample, two-tailed t-tests (i.e. the difference scores of two given conditions were tested against zero), and corrected for multiple comparisons.

For multivariate pattern analyses, we trained linear support vector machines (Fan et al., 2008) to distinguish voxel response patterns of congruent and incongruent conditions. Beta estimates from voxels of a given ROI were combined to form vectors of brain responses. For every run and every voxel, the time series of beta estimates was quadratically detrended to filter out low-frequency noise and subsequently *z*-scored. To make our analysis more robust against outliers, we set all values with a difference of >2 SD from the mean to −2 and 2, respectively. Classification was performed using cross-validation across runs in an odd/even fashion. Statistical significance of classification accuracies was evaluated using permutation tests. For every time we trained a classifier to discriminate between fMRI patterns, we refit new classifiers after randomly permuting the labels in the training set 10^4 times. We used the 10^4 classification accuracies from each observer to obtain a null distribution of mean accuracies at the group level expected under the null hypothesis (including the accuracy that was actually observed using the un-permuted dataset). From these null distributions, p values for a one-tailed test can be calculated as the number of values in the distribution that exceeded the observed accuracies divided by the number of permutations. We controlled the family-wise error (FWE) by constructing a common null distribution by taking the maximum value across ROI group means in each permutation step while making sure that the same label permutations were used in every ROI (Nichols and Holmes, 2002). The resulting null distribution was then used to calculate FWE-corrected p values. MVPA-Analysis were implemented using in-house code and the Princeton-MVPA toolbox (https://github.com/PrincetonUniversity/princeton-mvpa-toolbox).

Supplemental References

Fischer E, Bulthoff HH, Logothetis NK, Bartels A (2012) Human areas V3A and V6 compensate for self-induced planar visual motion. Neuron 73:1228-1240.

Nau M, Schindler A, Bartels A (2018) Real-motion signals in human early visual cortex. Neuroimage.

Petro LS, Vizioli L, Muckli L (2014) Contributions of cortical feedback to sensory processing in primary visual cortex. Front Psychol 5:1223.

Pitzalis S, Fattori P, Galletti C (2012) The functional role of the medial motion area V6. Front Behav Neurosci 6:91.

Pitzalis S, Fattori P, Galletti C (2015) The human cortical areas V6 and V6A. Vis Neurosci 32:E007.

Pitzalis S, Sereno MI, Committeri G, Fattori P, Galati G, Patria F, Galletti C (2010) Human v6: the medial motion area. Cereb Cortex 20:411-424.

Pitzalis S, Galletti C, Huang RS, Patria F, Committeri G, Galati G, Fattori P, Sereno MI (2006) Wide-field retinotopy defines human cortical visual area v6. J Neurosci 26:7962-7973.

Schindler A, Bartels A (2018) Integration of visual and non-visual self-motion cues during voluntary head movements in the human brain. Neuroimage 172:597-607.

Smith AT, Wall MB, Thilo KV (2012) Vestibular inputs to human motion-sensitive visual cortex. Cereb Cortex 22:1068-1077.
